# Anaerobic reduction of europium by a *Clostridium* strain as a strategy for rare earth biorecovery

**DOI:** 10.1038/s41598-019-50179-z

**Published:** 2019-10-04

**Authors:** Maleke Maleke, Angel Valverde, Alba Gomez-Arias, Errol D. Cason, Jan-G Vermeulen, Liza Coetsee-Hugo, Hendrik Swart, Esta van Heerden, Julio Castillo

**Affiliations:** 1 Department of Microbial, Biochemical and Food Biotechnology, University of the Free State, Bloemfontein, Republic of South Africa; 2 Institution of Groundwater Studies, University of the Free State, Bloemfontein, Free State Republic of South Africa; 3 Department of Physics, University of the Free State, Bloemfontein, Republic of South Africa; 4 iWATER solutions, Bloemfontein, Republic of South Africa

**Keywords:** Applied microbiology, Biogeochemistry, Environmental sciences

## Abstract

The biorecovery of europium (Eu) from primary (mineral deposits) and secondary (mining wastes) resources is of interest due to its remarkable luminescence properties, important for modern technological applications. In this study, we explored the tolerance levels, reduction and intracellular bioaccumulation of Eu by a site-specific bacterium, *Clostridium* sp. 2611 isolated from Phalaborwa carbonatite complex. *Clostridium* sp. 2611 was able to grow in minimal medium containing 0.5 mM Eu^3+^. SEM-EDX analysis confirmed an association between Eu precipitates and the bacterium, while TEM-EDX analysis indicated intracellular accumulation of Eu. According to the HR-XPS analysis, the bacterium was able to reduce Eu^3+^ to Eu^2+^ under growth and non-growth conditions. Preliminary protein characterization seems to indicate that a cytoplasmic pyruvate oxidoreductase is responsible for Eu bioreduction. These findings suggest the bioreduction of Eu^3+^ by *Clostridium* sp. as a resistance mechanism, can be exploited for the biorecovery of this metal.

## Introduction

Rare earth metals are critical raw material for the development of modern technological products due to their magnetic, photo physical, fluorescent, spectroscopic and luminescent properties^1–3^. For instance, luminescent europium (Eu) is used in magnetic resonance imaging^4^. Consequently, the demand for rare earth metals has increased rapidly^5^ making the acquisition of a stable and reliable supply of rare earth metals, in particular Eu, a top priority worldwide. Rare earth metals are commonly found in low concentrations in both primary (e.g., small deposits) and secondary (e.g., rock dumps and tailings) deposits^6–8^. However, despite low concentrations, the large amounts of mining and industrial waste make these deposits an attractive source for rare earth metals^9,10^.

In these environmental settings, site-specific microbes (e.g., *Clostridium*) are often exposed to metals without ‘adverse’ effects^11^. The activity of these bacterial species not only influences physiochemical parameters (i.e., pH, redox potential and ionic strength) but also contributes to the fate of metals and metal speciation (e.g., bioreduction and biomineralization)^12^. For example, a *Clostridium chromiireducens* strain isolated from a chromate contaminated site demonstrated the ability to directly reduce Cr^6+^ and indirectly reduce Fe^3+^ via electron shuttles^13^. Furthermore, several *Clostridium* strains have the ability to reduce and precipitate precious metals (i.e., Pd and Cu) as insoluble reduced compounds, bio-Pd (Pd^0^) and CuNP^14,15^. This feature can been exploited for biorecovery of precious metals in bioreactors under anaerobic conditions^16^.

The reduction of most rare earth metals was thought to be thermodynamically unfavourable, as they remain in the +3 oxidation state under different environmental conditions^17,18^. However, Eu can exist as +2 and +3^19–21^. In aqueous solution, the geochemical behaviour, bioavailability and speciation of Eu, like most transition metals, is mainly controlled by pH, oxidation potential (Eh) and temperature^20,22–24^. Therefore, under oxygen-limiting conditions bacterial metabolic processes, such as anaerobic respiration, could play a role in the speciation of Eu. The selective reduction of Eu^3+^ to Eu^2+^ could be useful for the separation of this metal due to the differences in chemical behaviour of Eu^2+^ compared to Eu^3+^ ^25^. Nonetheless, very little information exists on how *Clostridium* species interact with Eu, especially we do not know how clostridia bioaccumulate Eu species. Here, we aimed to investigate the ability of a *Clostridium* strain (*C*. sp 2611), isolated from sediments containing rare earth metals, to anaerobically reduce Eu^3+^ as a metal tolerance mechanism.

## Results

### Hydrogeochemistry of pore water

The pore water was characterized by near neutral pH (7.38) and microaerophilic (DO: 1.84 mg/L) conditions. The latter was also corroborated by the Eh value (99.9 mV). Total dissolved solids (2899 mg/L) and EC (419 mS/cm) values indicated high sulphate and metal concentrations. These concentrations were (in decreasing order): SO_4_ (3.2 M) > Mg (22.63 mM) > Na (11.27 mM) > Ca (11.03 mM) > Fe (1.80 µM). Rare earth metal concentrations contained in the pore water, in descending order, were: Sc (1.73 mM) > Pr (0.49 µM) > Ce (0.48 µM) > Sm (0.43 µM) > Yb (0.30 µM) > Tb (0.29 µM) > Nd (0.24 µM) > La (0.24 µM) > Gd (0.21 µM) > Ho (0.20 µM) > Lu (0.16 µM) > Eu (0.16 µM).

### Tolerance to trivalent europium

**Figure 1:**
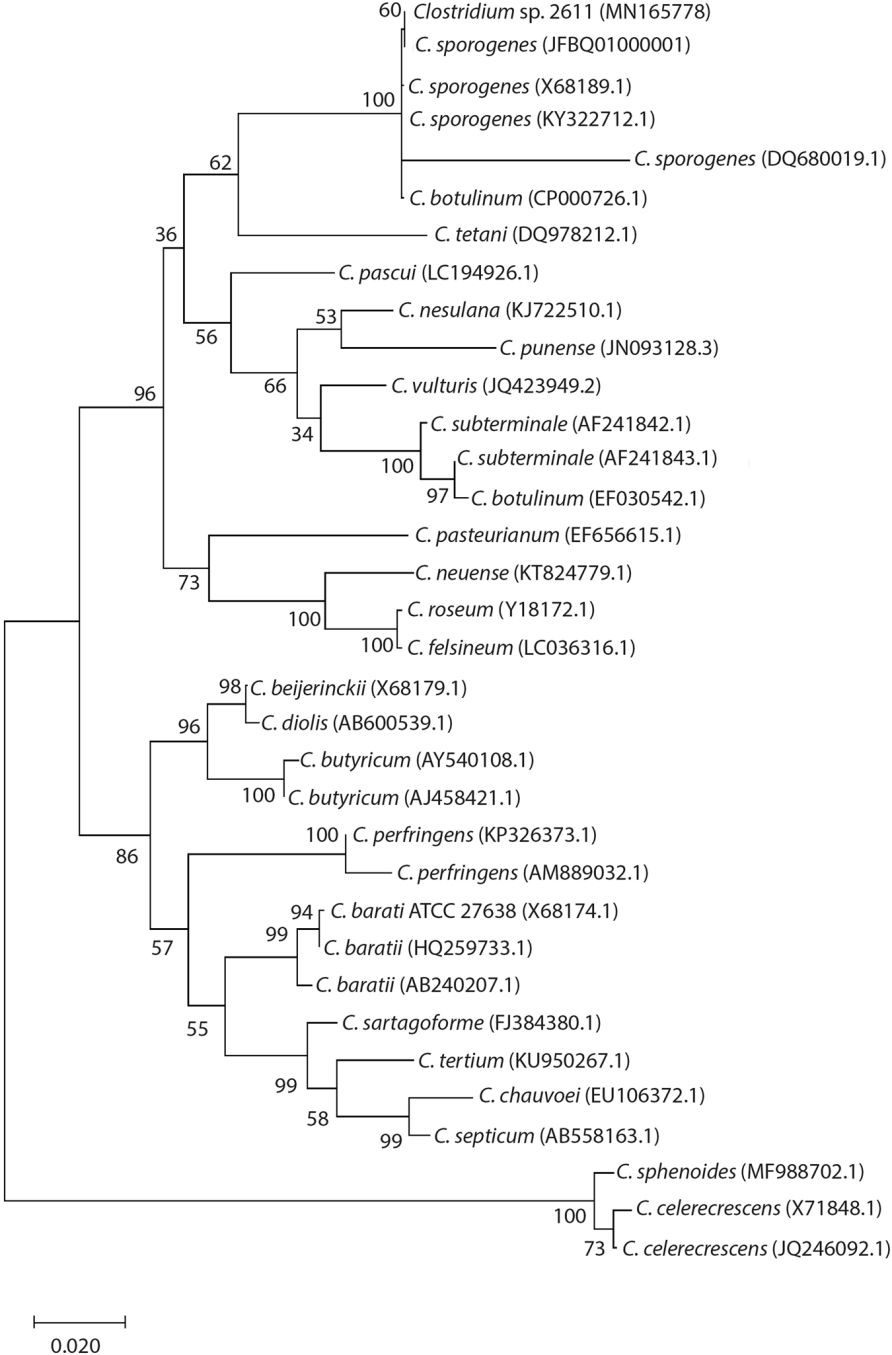
Phylogenetic tree based on a maximum likelihood analysis of partial 16S rRNA gene sequences showing the position of *Clostridium* sp. 2611 and the type strain of related species of Clostridia. Bootstrap values were obtained with the maximum-likelihood/minimum-evolution/neighbour-joining methods based on 1000 replicates.

**Figure 2:**
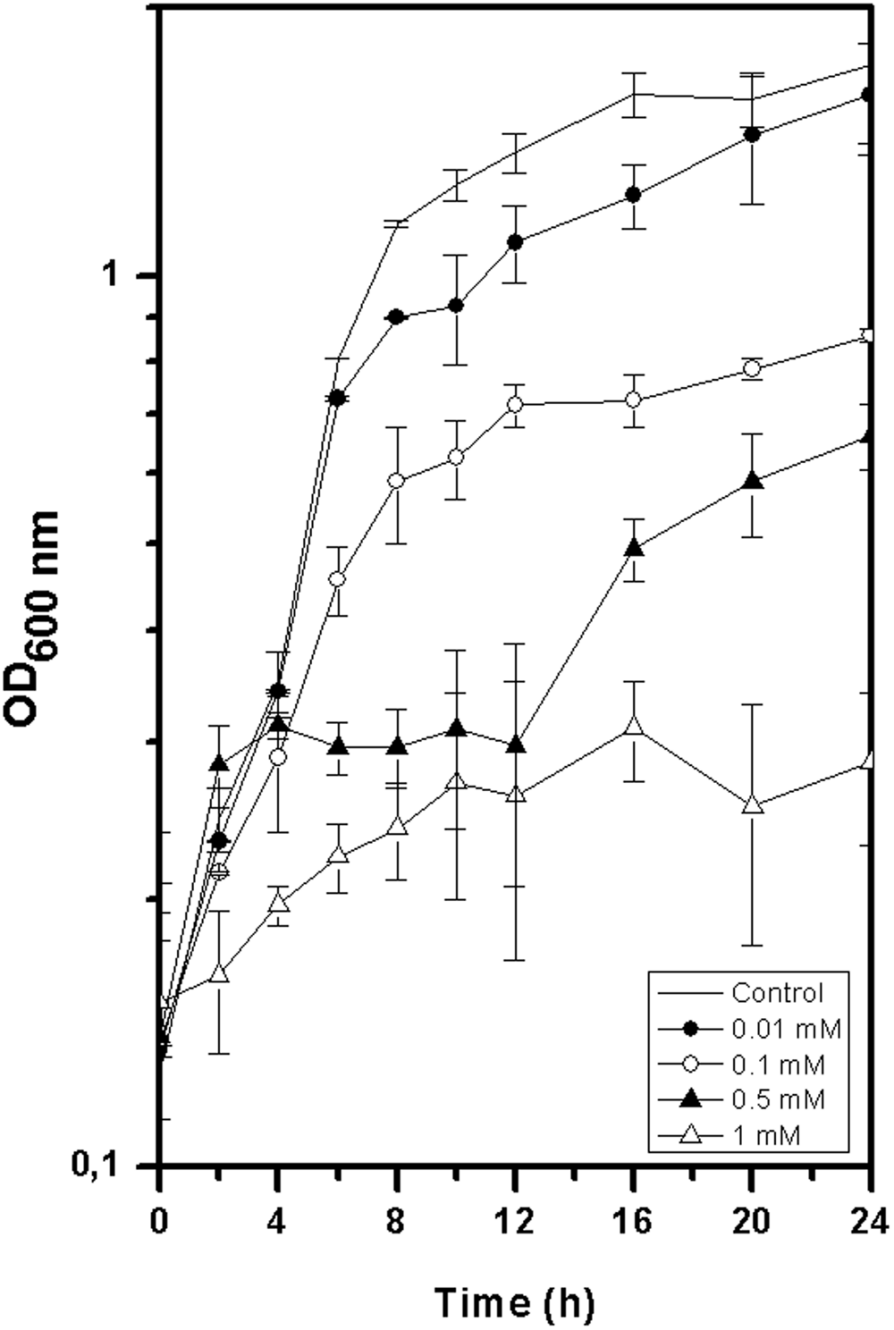
Growth of *Clostridium* sp. 2611 with europium in mineral salt media. Symbols indicate the mean value of OD_600nm_ samples. Error bars indicate standard deviations of samples.

*Clostridium* sp. 2611 (closely related to *Clostridium sporogenes* DSM 795 ^T^, 16S rDNA sequence identity of 99.85%, Fig. 1) could grow in mineral salt medium supplemented with up to 0.5 mM of Eu^3+^ (Fig. 2). In fact, at concentrations of 0.5 mM the bacterium showed an extended lag phase, followed by an exponential growth phase after 12 h. Overall, concentrations above 0.01 mM caused a decrease in microbial biomass when compared to the control (without Eu^3+^).

### Removal of trivalent europium

**Figure 3:**
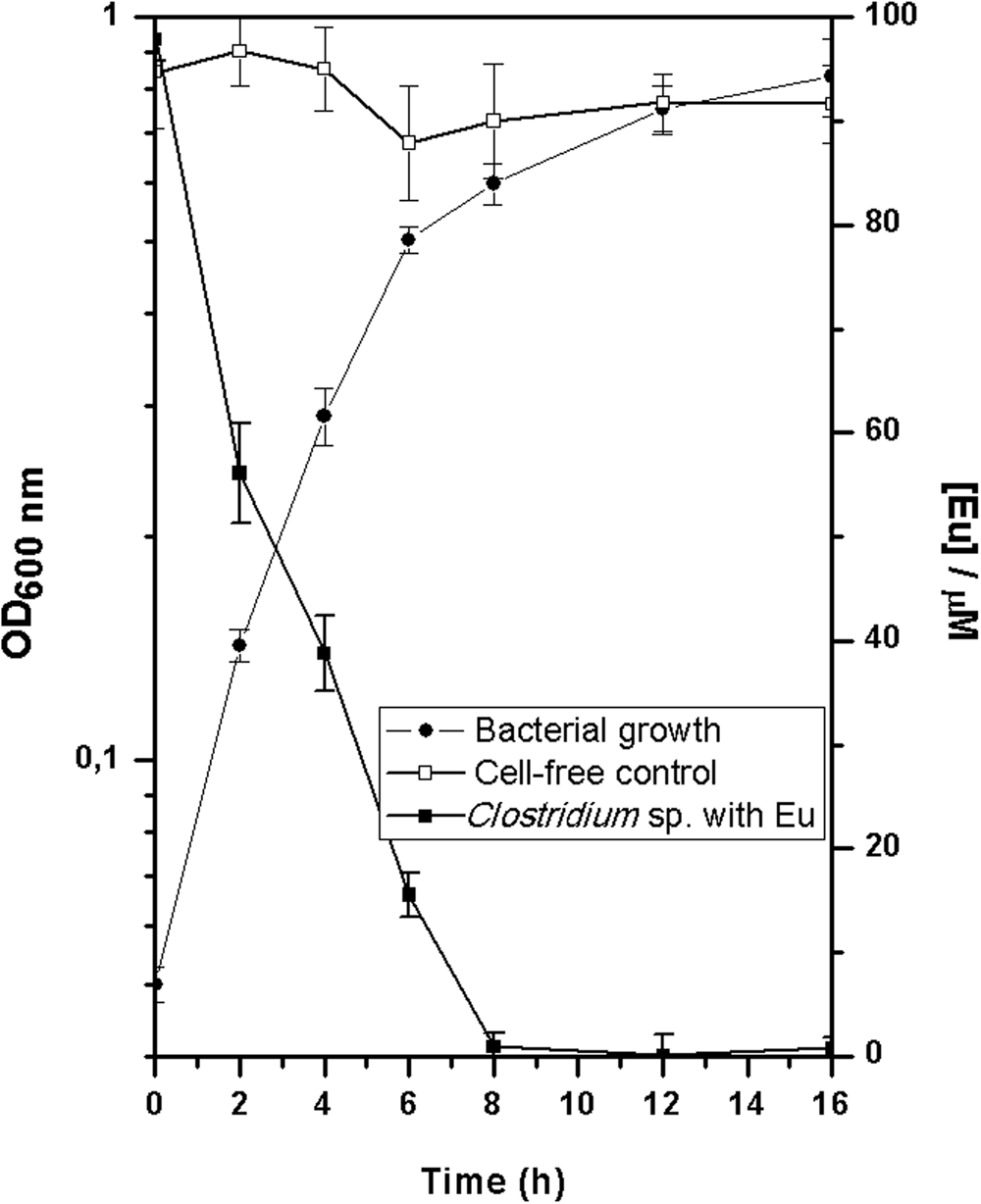
Growth of *Clostridium* sp. 2611 with europium in mineral salt media. Symbols indicate the mean value of OD_600nm_ samples, while standard deviations indicated by error bars.

Complete removal of Eu^3+^ (0.1 mM) from the culture medium was observed within 8 h of growth (Fig. 3). Europium precipitation did not take place in the abiotic (cell-free) controls, which suggests that the removal of Eu^3+^ is biologically driven.

### Electron microscopy

**Figure 4:**
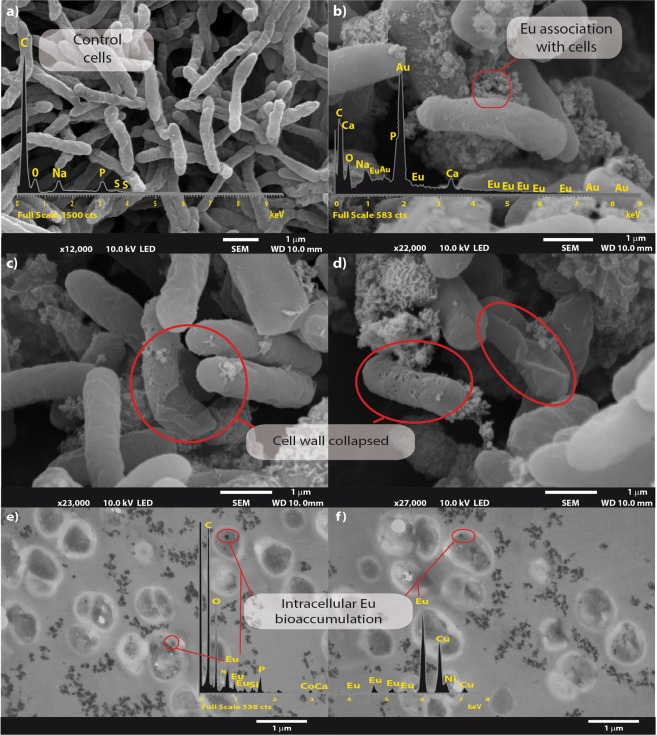
Electron microscopy micrographs of *Clostridium* sp. 2611. Bars indicate the scale as micrometres and red shapes indicates association of Eu. (**a**) Scanning electron microscope of *Clostridium* sp. control cells, and (**b**) Eu^3+^ exposed cells. Europium damaged cell wall. (**c**,**d**) Transmission electron microscopy micrographs of Eu^3+^ bioaccumulation by *Clostridium* sp. (**e**,**f**), red circles indicate the location of Eu (Shown as electron-dense granules). Overlaid EDX spectra.

Scanning electron microscopy of *Clostridium* sp. 2611 cells depicted typical rod-shaped morphology with rough surfaces (Fig. 4). In addition, the micrographs of cells exposed to Eu^3+^ showed that the cell wall collapsed at concentrations up to 0.1 mM (Fig. 4c,d). Electron dispersion X-ray (EDX) spectroscopy analyses identified extracellular amorphous precipitates, composed primarily of Ca, Eu, PO_x_ and CO_x_ on the cell surface. The intense Au peak resulted from the gold coating during sample preparation. Conversely, TEM analysis did not show cell surface accumulation of Eu but rather intracellular accumulation (Fig. 4e,f). Energy dispersive X-ray analysis spectra confirmed that the black precipitates inside and outside the cells were mostly composed of Eu and phosphate.

### Reduction of trivalent europium

**Figure 5:**
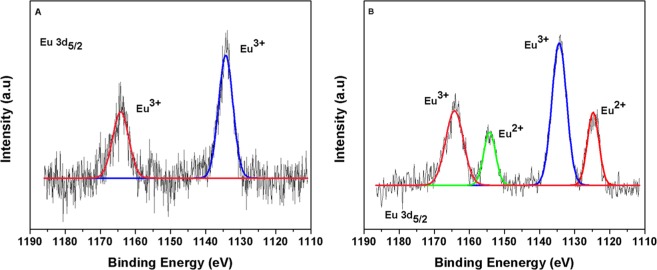
High-resolution X-ray photoelectron spectroscopy of (**A**) cell-free control and (**B**) reduction of Eu^3+^ in mineral salt media amended with *Clostridium* sp. 2611.

The HR-XPS analysis demonstrated bioreduction of Eu^3+^ to Eu^2+^ by *Clostridium* sp. 2611. The fitted spectra curve shows four major peaks (1123.8, 1133.6, 1154.4 and 1163.7 eV) (Fig. 5B). The 1123.8 and 1154.4 eV peaks were identified as divalent Eu^19,21,26,27^. The binding energy demonstrate a mixture of Eu^2+^ and Eu^3+^ under growth conditions.

**Figure 6:**
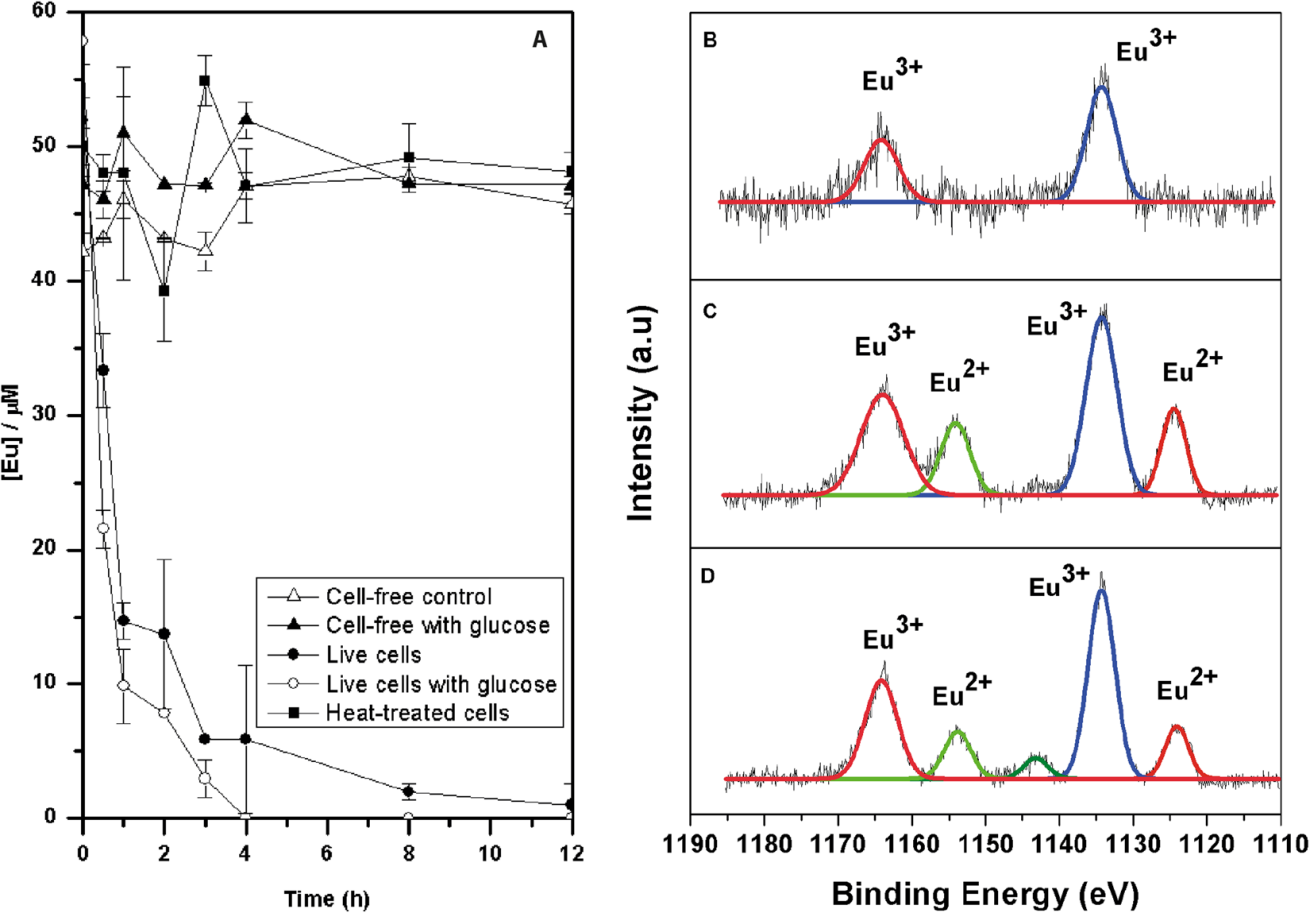
(**A**) Profile of the reduction of Eu^3+^ by resting cells. High resolution XPS spectrum of (**B**) cell-free control, (**C**) live cells with glucose as electron donor, and (**D**) live cells without electron donor.

Under non-growth conditions live cell suspensions, with or without glucose as an electron donor, removed up to 100% of Eu^3+^ (Fig. 6A). It is worth mentioning that no chemical removal or complexation was observed in the cell-free suspensions and glucose experiment without cell suspensions (Fig. 6B). The HR-XPS analysis demonstrated bioreduction of Eu^3+^ to Eu^2+^ under non-growth conditions (Fig. 6C,D).

### Localization and identification of proteins

The separation of the different subcellular fractions demonstrate weak Eu^3+^ reducing activity in the periplasmic fraction (6.1 µM Eu^3+^ mg^−1^ protein at 6.7% total Eu^3+^ loaded), and the addition of electron donors had no effect on how fractions interact with Eu^3+^ (50 µM) (Table 1). The membrane fraction could remove 120 µM Eu^3+^ mg^−1^ protein h^−1^ followed by cytoplasmic fraction 70.48 µM Eu^3+^ mg^−1^ protein h^−1^, which represents 79.7% and 76.4% total Eu loaded, respectively. However, it is worth noting that chemical removal or complexation was observed in the heat-treated membrane fraction with similar levels of activity to the non-treated fraction probably due to cellular debris, indicating abiotic removal^28^. Table 1 summarizes a comparison of Eu^3+^ interaction with the different cellular fractions of *Clostridium* sp. 2611 following exposure to 50 µM of Eu^3+^.

**Table-1:** Localization of the anaerobic Eu reductase activity.

Fractions	Non-treated*	Heat-treated*
Periplasmic	6.1 ± 2.3	1.4 ± 0.9
Membrane	120.8 ± 5.7	117.4 ± 6.1
Cytoplasm	70.4 ± 3.6	13.2 ± 4.3

*Activity expressed as µM. mg protein per hour.

**Figure 7:**
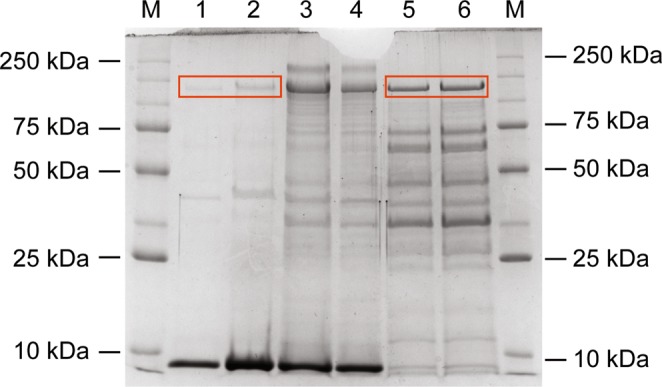
SDS-PAGE of the different subcellular fractions. Lanes M: molecular marker, Lanes 1: Periplasmic fraction, Lane 2: Periplasmic fraction with Eu, Lanes 3: Membrane fraction, Lane 4: Membrane fraction with Eu, Lanes 5: Cytoplasm fraction, Lane 6: Cytoplasm fraction with Eu. Red box indicates pyruvate flavooxidoreductase.

After separation of the different subcellular fractions of *Clostridium* sp. 2611 with and without Eu^3+^, slight changes in the protein profiles were observed in the presence of Eu^3+^ in both periplasmic and cytoplasmic fraction (Fig. 7). Since various proteins can be approximately the same size it is very unlikely that only one band will be excised from a SDS-PAGE gel. There seems to be changes in the proteome profile at a protein approximately 130 kDa (MW) in lanes 2 and 6, respectively identified as flavooxidoreductase. While, approximately a 45-kDa protein showed changes in the periplasmic fraction (flagellin). Furthermore, slight changes are observed in the cytoplasmic fraction with Eu^3+^ (lane 6). There are two proteins approximately of 45 and 30 kDa, glucose-6-phosphate isomerase and triosephosphate isomerase respectively. Differences could not be detected in the membrane fractions (lanes 3 and 4).

## Discussion

In general, heavy metal exposure promotes the emergence of resistance mechanisms such as reductive precipitation in bacteria^29,30^. Interestingly, different clostridia have demonstrated metal bioreduction and biomineralization capabilities^15,31^. Here we explored the tolerance mechanism of *Clostridium* sp. 2611 to Eu^3+^. We observed that Eu^3+^ is detrimental to the cell at concentrations above 0.1 mM, which is in agreement with the findings of Kurvet *et al*.^32^, who reported that high concentrations (between 0.025 and 0.17 mM) of rare earths (La^3+^, Ce^3+^, Pr^3+^, Nd^3+^ and Gd^3+^) were toxic to the marine bacterium *Vibrio fischeri*. It was postulated that the toxicity of these metals was related to the comprised integrity of the cellular membrane. Lack of integrity of the membrane of *Clostridium* sp. 2611 was confirmed by FE-SEM analysis (Fig. 4c,d).

The biological removal of Eu^3+^ was maximal during exponential growth phase, probably due to the higher cellular activity, as was demonstrated previously for *Thermus scotoductus* SA-01^33^. Using FE-SEM-EDX analysis it was observed that the external Eu^3+^ precipitates were neoformed mineral complexes [Ca_x_^+^Eu_x_^+^(CO)_y_]_n_ likely due to the indirect effect of the glucose fermentation by *Clostridium* sp. 2611^34–36^, although active mechanisms cannot be excluded. Usually, the excretion of metals is due to cellular homeostasis to maintain optimal internal conditions for metabolism and energy transduction, as it was reported for *Clostridium* sp. interacting with U^6+^ ^37–39^. Therefore, it needs to be further investigated whether the precipitates surrounding the cell resulted as a consequence of active and/or passive mechanisms. Transmission electron microscopy micrographs showed intracellular accumulation of Eu. The intracellular Eu could be immobilized as inorganic polyphosphates (PolyP), which seem to be in the cell according to TEM-EDX analysis^40,41^. Inorganic PolyP has been shown to accumulate Hg, As, Cu, and Ni^42^, which suggests that PolyP might be used by microorganisms for storing different metals, including Eu.

In general, the intracellular accumulation of metals results from active transport^43^ as it was observed, for example, in *Shewanella* sp. HN-41 accumulating reduced uranium^44^. Certainly, the HR-XPS results indicate that *Clostridi*um sp. 2611 can reduce Eu^3+^ to Eu^2+^, which could facilitate its transport to the cytoplasm. To the best of our knowledge, this is the first report showing the reduction of Eu^3+^ by a bacterium, implying an association between bacterial growth demands and Eu. Under anaerobic conditions, bacteria can employ reductive mechanisms to generate energy^45–48^. For example, *Desulfotomaculum reducens* MI-1 is able to use different metals (i.e., Cr, U, Fe and Mn) as final electron acceptors for growth^48^.

The metal reduction commonly observed in bacterial cultures may be due to direct (bacterial activity), indirect (electron shuttle) and abiotic reduction (due to media constituents), which are not mutually exclusive. For instance, all these mechanisms have been observed in *Desulfovibrio alaskensis* G20^49^. In this study, non-growing conditions were selected to exclude metal reduction from growth-related cellular processes and stimulate competition for limited substrates^50,51^, as the only electron acceptor was Eu^3+^. Europium reduction was observed with and without external electron donor (i.e., glucose). This has been reported different metals in other metal-reducing bacteria, including *Cellumonas* sp., *Deinococcus* *radiodurans* R1, and *T*. *scotoductus* SA-01^52,53^. In the absence of exogenous electron donors, reduction of metals most likely results from internal electron donors^52^ such as glycolipids.

The fact that similar levels of Eu^3+^ removal were observed in the membrane fraction and the abiotic control, suggests that membrane proteins do not play a role in Eu^3+^ reduction. Cellular debris such as polysaccharides and lipids are capable of complexing metals in solution^54,55^ and might explain these results. In contrast, according to our results, proteins like pyruvate flavodoxin oxidoreductase appear to be involved in Eu^3+^ reduction. However, we cannot determine at this point if the reduction of Eu^3+^ occurs in the periplasm or in the cytoplasm. In some methylotrophic bacteria, intracellular transport of Eu induces the activity of methanol dehydrogenases^56–60^. On the other hand, divalent metals such as Ca^2+^, an analogue of Eu, play a role in enzyme activity and stability as demonstrated for *Bacillus stearothermophilus*^61^. Overall, these results suggest that *Clostridium* sp. 2611 might use Eu to conserve energy or as co-factor for different enzymes.

In summary, here we isolated a site-specific *Clostridium* (*C*. sp 2611 closely related to *C*. *sporogenes* DSM 795 ^T^) able to interact with Eu. This bacterium displayed several resistance mechanisms against Eu^3+^, i.e., bioreduction and intracellular accumulation, which have biotechnological potential to recover rare earth Eu from mining waste. This discovery far extends our knowledge of microbial rare earth metal interactions beyond passive sorption.

## Methods

### Source of bacterium

The sampling took place in a stream in the vicinity of the Phalaborwa Complex. The complex is located in North-Eastern Limpopo region (South Africa) and mines a world unique deposit of copper carbonate^62,63^. The mining of copper generates large amounts of carbonatite rock waste with significant rare earth concentrations^64,65^ that leach into nearby streams. Stream sediment was collected aseptically in degassed 150 ml sterile serum bottles with Teflon-coated septa (Wheaton, USA). Glass serum bottles with zero headspace were kept upside-down at 4 °C and transported to the laboratory within 24 h. Sediments were centrifuged at 2000 × *g* for 5 min and pore water samples (10 ml) collected in an anaerobic chamber (COY Laboratory Inc., USA) and stored at 4 °C.

### Hydrogeochemical characterization

Temperature, pH, oxidative reduction potential (ORP), electrical conductivity (EC) and dissolved oxygen (DO) were determined on-site with a HI 9828 pH/ORP/EC/DO probe (Hanna Instrumentations, USA). ORP measurements were corrected to the standard hydrogen electrode^66^. Total elemental composition (including rare earth concentrations) was determined by ICP-MS (Perkin Elmer, USA).

### Enrichment and isolation

Anaerobic microorganisms were enriched using liquid solution containing sediment (10% w/v) and anaerobic dilute heterotrophic media (per liter): Glucose 0.1 g, Yeast extract 0.1 g, Peptone 0.05 g, Tryptone 0.05 g, MgSO_4_.7H_2_O 0.6 g, CaCl_2_.2H_2_O 0.07 g, MOPS 0.1 g, 100 µL mineral and 100 µL vitamin solution. The enrichment culture was incubated at room temperature under strict anaerobic conditions. In order to isolate a *Clostridium* strain, serial dilutions were grown on petri dishes using reinforced *Clostridial* medium (Difco laboratories Inc, US). Single colonies were suspended anaerobically (Coy anaerobic chamber) in *Clostridium* 54b medium (https://www.dsmz.de) and incubated for 24 h at 37 °C.

### Taxonomic identification of the isolate by 16S rRNA gene sequence analysis

Total gDNA of a *Clostridium* isolate was extracted using the NucleoSpin® Kit for Soil (Macherey-Nagel, Germany) as per manufacturer’s instructions. 16S rRNA gene sequencing was performed using universal primers^67^. DNA solution (10 ng template DNA final concentration), was added to the PCR mixture containing, 0.75 µl of each primer (10 µM), 12.5 µl of 2 × KAPA HiFi HotStart ReadyMix and ultrapure Milli-Q to dilute the total volume to 25 µl. PCR conditions were as follows: initial DNA denaturation for 3 min at 95 °C, 25 amplification cycles (20 sec at 98 °C, 15 sec at 58 °C, 45 sec at 72 °C) and final primer elongation for 1:30 min at 72 °C. The 16S rRNA sequence has been deposited in GenBank (accession number: MN165778). Taxonomic identification was performed using EzBioCloud (https://www.ezbiocloud.net).

### Standard cultivation conditions

Unless otherwise stated, *Clostridium* sp. 2611 was cultivated under anaerobic conditions in mineral salt media containing (per litre): glucose 5.0 g, NH_4_Cl 0.5 g, glycerol phosphate 0.3 g, MgSO_4_.7H_2_O 0.2 g, CaCl_2_.2H_2_O 0.5 g, peptone 0.1 g, yeast extracts 0.1 g and FeSO_4_.7H_2_O 2.8 mg (pH 7.0) at 30 °C for 24 h. The medium was dispensed in 150 ml serum bottles purged with O_2_-free N_2_, pressurized at approximately 80.0 kPa for 60 min, and sealed with aluminum seals before autoclaving.

### Tolerance to trivalent europium

The *Clostridium* strain was cultivated to mid-exponential growth phase (OD_600nm_ = 0.6). Five ml of a standardized cell suspension was used to initiate growth in fresh mineral salt media (95 mL in serum bottles) containing EuCl_3_.6H_2_O as Eu^3+^ (0, 0.01, 0.1, 0.5, 1 mM) and grown for 24 h. The bacterial growth was monitored spectrophotometrically (OD_600nm_) at 2 h intervals. The experiments were performed in triplicate.

### Removal of trivalent europium

Bacterial cultures were exposed to 0.1 mM of Eu^3+^, based on tolerance assays, in fresh mineral salt media. Optical density (OD_600nm_) and removal of Eu^3+^ were monitored for 24 h at 2 h intervals. Europium^3+^ removal from the media was monitored using the arsenazo-III method^68^ as described by Maleke *et al*.^69^.

### Electron microscopy

*Clostridium* cells were harvested by centrifugation (6000 × *g*; 10 min; 4 °C) and portions of the pellet were characterized by microscopic techniques. The morphology and the semi-quantitative analysis of the extracellular precipitates were determined using JSM-7800F thermal field emission scanning microscope coupled with Oxford Aztec 350 × -Max80 Energy-Dispersive X-ray (EDX) analysis (Oxford Instruments, UK). A portion of the cells were fixed in 2.5% glutaraldehyde and rinsed with 0.1 M phosphate buffer (pH 7.0). Thereafter, the cells were treated in a series of escalating ethanol (50, 70, 90, and 95%) concentration for 30 min per step. This was followed by two absolute ethanol dehydration steps performed for 1 h each. The cells were critical point dried, mounted on metal stubs, coated with gold and analysed. Intracellular bioaccumulation was observed and semi-quantitatively analysed by Transmission Electron Microscopy (TEM). Dehydrated cells (method described in the analysis by FE-SEM) were embedded in epoxy resin and polymerised at 70 °C for 8 h. Thin sections (0.2 µm) were cut using an ultra-microtome UM7 (Leica Microsystems, Germany) and collected on copper grids for the analysis. Transmission electron micrographs were taken with a Philips CM100 (FEI, USA) coupled with an Oxford X-ray analyser coupled with energy dispersive X-ray (EDX) spectrum (JSM-7800F) (Oxford Instruments, UK).

### Europium speciation determination

Europium oxidation/valence state was determined using high-resolution x-ray photoelectron spectroscopy (HR-XPS) according to Maleke *et al*.^69^. Briefly, *Clostridium* cells were harvested by centrifugation (6000 × *g*; 10 min; 4 °C) following standard incubation conditions with 0.1 mM Eu^3+^. The pellets were lyophilized, embedded on a carbon tape and analysed in a vacuum chamber. HR-XPS results were acquired using a PHI 5000 Versaprobe system (Physical Electronics, USA) as described by Yagoub *et al*.^70^.

### Reduction of trivalent europium under non-growth conditions

Resting cells were prepared using standard cultivations conditions (without Eu^3+^ added). Cells were harvested by centrifugation (6 000 × *g*; 15 min; 4 °C), the supernatant discarded, and the cell pellets washed in fresh 20 mM 1, 3-(N-morpholino) propane sulfonic acid (MOPS; pH 7.0) buffer. This process was performed three times and the cells were then re-suspended in 10 ml of MOPS buffer. 500 mg of wet biomass were added to a solution containing 50 µM Eu^3+^ in anaerobic MOPS buffer, pH 7.0, with 10 mM glucose to assess. Timed samples (16 hours at 2 h intervals) were aseptically withdrawn and centrifuged immediately (6000 × *g*, 10 min; 4 °C) and the supernatant analysed for the decrease of Eu^3+^. Cell-free controls, donor free and heat-treated cells were used in parallel to the live-cell experiments to assess abiotic Eu^3+^ reduction. The entire treatment of the cells was performed inside an anaerobic chamber (atmosphere of N_2_–H_2_, 95:5; Coy Laboratory Inc., USA). The ability of the *Clostridium* isolate to reduce Eu was determined by HR-XPS analysis following standard procedure described above.

### Cellular preparation

Subcellular fractions were prepared under anaerobic conditions using the methodology described by Gaspard *et al*.^71^. Briefly, to extract the periplasmic fraction, 20 mM MOPS-NaOH buffer (pH 7.0) was used to wash twice a pellet of *Clostridium* strain cells cultivated under standard conditions. Approximately 1 g (wet weight) cells were resuspended in 20 ml of buffer containing 25% (w/v) sucrose. The cellular wall was degraded using 0.1% (w/v) Lysozyme and cellular disruption was performed using 5 mM EDTA, pH 8.0 and 13 mM MgCl_2_. Finally, the spheroplast were separated from the periplasmic fraction by centrifugation (20 000 × *g*; 30 min; 4 °C).

The membrane and cytoplasmic fractions were obtained from spheroplast cells treated with 10 ml of 20 mM MOPS-NaOH buffer (pH 7.0), DNase crystals and EDTA-free protease inhibitor cocktail (Roche, Germany) and disrupted by ultrasonic treatment (6 repeats, 100 W, 30 s on ice) using a Branson Sonic Power Sonifier Cell Disruptor B-30 (Danbury, USA). The crude extract was separated from cell debris by centrifugation (4000 × *g*; 10 min; 4 °C), while cytoplasmic fraction containing soluble proteins (supernatant) and a membrane fraction were obtained (pellet) by centrifugation (100 000 × *g*; 90 min; 4 °C). Finally, 20 mM MOPS−NaOH buffer (pH 7.0) was used to resuspend the membrane fraction as described by Maleke *et al*.^69^. Total protein concentrations for each fraction were determined with a bicinchoninic acid assay kit from Pierce (Rockford, USA) by the method of Smith *et al*.^72^, using bovine serum albumin (BSA) as a standard.

### Localization of trivalent europium reduction activity

Europium reduction activity was assayed in each of the cellular fractions by monitoring the decrease of Eu^3+^ using the arsenazo-III method. The reaction mixture contained 50 µM of Eu^3+^, 10 mM NADPH or NADH as electron donor in anaerobic MOPS buffer (pH 7.0). The assay was initiated by adding subcellular fraction extracts of *Clostridium* sp. 2611 to the reaction mixture. The experiments were performed at 37 °C in an anaerobic chamber (COY Laboratories Inc., USA). Fraction-free controls and heat-treated fraction controls were used to assess abiotic Eu^3+^ reduction. The heating of the fractions^73,74^ was done in order to assess the reduction of Eu^3+^ by organic matter.

### Identification of proteins involved in trivalent europium reduction

SDS-PAGE was performed as described by Laemmli^75^, using a SE 200 gel electrophoresis unit (Hoefer Scientific Instruments, USA), to assess changes in protein profiles in the presence of Eu^3+^. Normalized 500 ng of total fraction protein for both control and Eu^3+^ treated samples were loaded and separated on a 12% resolving gel with a 4% stacking gel. Protein profiles were visualized by Coomassie staining. After separation, differential protein bands were excised, subjected to tryptic in-gel digestion and identified by liquid chromatography–mass spectrometry using an AB SCIEX 4000 spectrometer (Shimadzu, Japan).

## Supplementary information

Gel original
