# Supplementary material for: Anaerobic reduction of europium by a Clostridium strain as a strategy for rare earth biorecovery
Source: Sci Rep. 2019 Oct 4;9:14339. doi: 10.1038/s41598-019-50179-z (PMC6778152; doi:10.1038/s41598-019-50179-z)

Anaerobic reduction of europium by a *Clostridium* strain as a strategy for rare earth biorecovery

Maleke Maleke[1], Angel Valverde[1], Alba Gomez-Arias[1,2], Errol D. Cason[1], Jan-G Vermeulen[1], Liza Coetsee-Hugo[3], Hendrik Swart[3], Esta van Heerden[4] and Julio Castillo[1*]

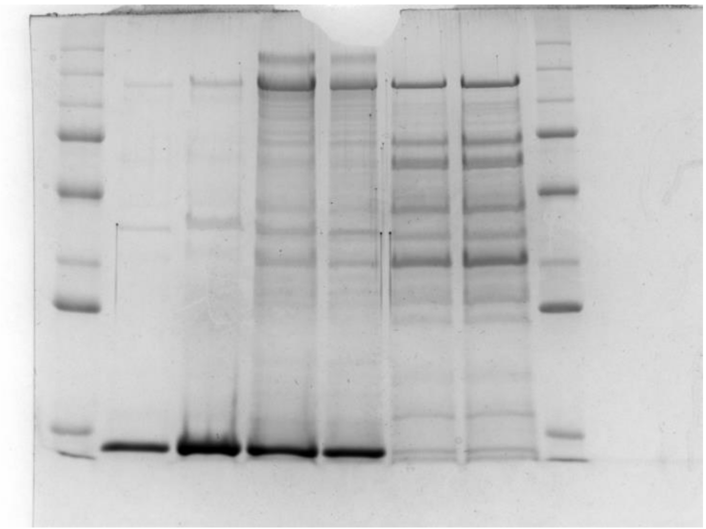

Supplement: Supplementary file 1 — Gel original [file 41598_2019_50179_MOESM1_ESM.pdf]
